# Translation and cross-cultural adaptation of Persian version of Evidence Based Medicine Questionnaire (EBMQ) in postgraduate medical students in Iran

**DOI:** 10.1371/journal.pone.0301831

**Published:** 2024-04-16

**Authors:** Ziba Danaei, Farzan Madadizadeh, Fatemeh Sheikhshoaei, Hossein Dehdarirad

**Affiliations:** 1 Department of Medical Library and Information Sciences, School of Allied Medical Sciences, Tehran University of Medical Sciences, Tehran, Iran; 2 Departments of Biostatistics and Epidemiology, School of Public Health, Shahid Sadoughi University of Medical Sciences, Yazd, Iran; American University of Beirut Medical Center, LEBANON

## Abstract

**Background:**

Evidence Based Medicine Questionnaire (EBMQ) was developed to assess the knowledge, practice and barriers towards the implementation of Evidence-Based Medicine (EBM). This study aimed to translate, cross-culturally adapt and psychometrically validate the Persian version of EBMQ.

**Methods:**

This was an analytical cross sectional study. The EBMQ underwent translation and cross-cultural adaptation following best practices. Face validity was assessed by a panel of five experts. The content validity index (CVI) and content validity ratio (CVR) were evaluated by 15 experts who were familiar with EBM. The tool’s internal consistency and test-retest reliability over a 2-week period were evaluated using Cronbach’s α and intra-class correlation (ICC), respectively. To assess construct validity, the questionnaire was completed by 400 medical students. Exploratory factor analysis (EFA) and confirmatory factor analysis (CFA) were used for construct validity assessment. All analyses were carried out using IBM SPSS v.24 and AMOS v.24. A significance level of 5% was considered.

**Results:**

The CVR for 40 items was higher than 0.62, and their CVI score was 1.0, indicating good content validity. The scale demonstrated acceptable internal consistency and test-retest reliability (n = 30) with an ICC of 0.909 (95% CI: 0.866 to 0.941), and an overall alpha coefficient of 0.957. The structural validity was established through exploratory factor analysis (Bartlett’s test p<0.001; the Kaiser-Meyer-Olkin index = 0.862), and it was further confirmed by confirmatory factor analysis. A three-factor solution with 40 items, explaining 51.610% of the variance, exhibited the best fit indices Chi-square statistics/df = 4.23; RMSEA = 0.08; CFI = 0.95; NFI = 0.93; TLI = 0.92).

**Conclusion:**

The Persian version of the EBMQ was a reliable and valid tool that could be utilized to assess the knowledge, practice and barriers of EBM for physicians in Persian language countries.

## Introduction

Over the course of four decades, the concept of Evidence-Based Medicine (EBM) has become widely accepted as a standard in practice and decision-making in healthcare systems [1]. EBM in health care follows a systematic approach to address clinical problems and is an effective approach in both clinical medicine education and practice [2,3]. The implementation of EBM has been shown to lead to improved patient outcomes, enhanced quality and safety of care, reduced healthcare costs, and improved overall practice [4,5]. Applying EBM allows physicians to access the most effective treatment methods and make clinical decisions based on the highest quality evidence available [6].

Learning and applying EBM among medical students provides them with an opportunity to practice EBM skills and promotes the use of evidence in making patient-care decisions [7]. In Iran, EBM education has been considered in the graduate curriculum in many medical schools and has received attention in many fields of medical sciences in recent years [8]. However, awareness of medical professionals in Iran about EBM-specific terms and databases, as well as the knowledge, attitude and practice of different groups is at a low level [9,10].

Several studies that aimed to determine the barriers and facilities of EBM in Iran have reported that the main barriers to applying EBM are the lack of sufficient knowledge and skills, adequate facilities, lack of time, unfamiliarity with research methods and lack of authority to make change [9,11,12]. The results of some studies revealed that, facilities such as creating opportunities, teaching research methods and holding training courses should be considered [10,13,14]. A valid and reliable tool is needed to evaluate EBM among health providers [15]. A systematic review in 2020 found that tools with reasonable validity are available for evaluating some domains of EBM [16]. Most instruments mentioned in that study assess knowledge, skill and attitude domains.

To the best of our knowledge, the majority of studies conducted in Iran concerning the status of attitudes, knowledge, practices, and the identification of barriers of implementing EBM have not utilized well-standardized and validated instruments. To gain a deeper understanding of the barriers to implementing evidence-based medicine, and to assess knowledge and practice, we require an updated, validated, and standardized tool. This tool will help identify gaps in knowledge and practice, and shed light on the barriers, thereby guiding future educational interventions and policy changes. Recognizing that developing a new tool can be time-consuming and costly, our study aims to translate and culturally adapt a standard tool for Persian-speaking physicians.

The EBMQ originally developed in Malaysia[17], is one of the available tools that is a valid and reliable instrument to assess the knowledge, practice and barriers toward the implementation of EBM. In the current research, we were looking for a tool, while focusing on knowledge and practice, and addressing the barriers to implementing EBM as well. Hence, the objective of this research is to undertake the translation and cross-cultural adaptation of the EBMQ into Persian, and to assess the validity and reliability of the Persian version of the EBMQ.

## Materials and methods

### Design

This study utilized an analytical cross-sectional research design to assess the validity and reliability of the Persian version of the EBMQ. The translation and cross-cultural adaptation of the tool were executed following recommended best practices [18,19].

### Research instrument

The 84-item EBMQ [17] which was published in English in 2018 consisted of 42 main items that were divided into three domains: knowledge, practice, and barriers. The knowledge domain included 16 items assessing knowledge of medical terms on the 5-point Likert scale (ranging from 1 never heard of this term before to 5 understand this term well and able to explain what it means to others). Additionally, there are 8 items evaluating knowledge of information sources on a 4 point Likert scale from 1 (unaware) to 4 (read and used in clinical decision making). The practice domain consists of 8 items with a 5-point Likert scale ranging from 1 (strongly disagree) to 5 (strongly agree). Lastly, the barriers domain comprises 10 items assessed on a 5-point Likert scale from 1 (strongly disagree) to 5 (strongly agree). Each question is scored according to the Likert scale, with a higher score indicating a better situation. Total scores are calculated for each domain, as well as an overall score.

### EBMQ translation and cross-cultural adaptation process

Permission was obtained from the copyright holder to translate the EBQM into Persian. For the translation, a forward-translation back-translation design was used based on recommended guidelines [18,20,21]. In the forward step, two bilingual translators, native Persian language with expertise in EBM, independently translated the questionnaire from English to Persian. The focus of the translation was on achieving conceptual and cultural equivalence, rather than a literal translation. An expert panel reviewed and made modifications to the two forward-translated versions of the instrument. The expert panel then agreed on a version for back-translation. In the back translation step, two other independent bilingual translators with native English language, who were not aware of the original English version, translated it back into English. The expert panel reviewed the compatibility of the Persian and English versions, after confirmation ultimately, the Persian version of the EBQM was applied to sample data.

### Participants

#### Sample size calculation

In this psychometric study, in the validity and reliability section, different sample sizes were considered, all of which were in accordance with the psychometric guidelines of the questionnaire [22]. 5 experts were used in face validity, 15 experts and qualified students were used in content validity. In the structural validity check, since our questionnaire (EBQM) had 40 items, according to the guidelines, we included 10 samples for each item, as a result, the sample size in this section was 400 students (According to number of students in each university, we determined that 149 samples should be collected from Tehran University of Medical Sciences, 150 from Shahid Beheshti University of Medical Sciences, and 101 from Iran University of Medical Sciences). 30 qualified students according to the guidelines were used in the stability reliability, and internal consistency.

#### Sampling technique

The sampling method for examining face and content validity was purposive sampling. The sampling method for examining construct validity and reliability was proportionate stratified sampling among the students of three universities of medical sciences in Iran (Tehran University of Medical Sciences, Shahid Beheshti University of Medical Sciences, Iran University of Medical Sciences).

#### Inclusion and exclusion criteria

The inclusion criterion for our study was medical students who were currently enrolled in internship, residency, specialty, fellowship, or subspecialty programs at Shahid Beheshti, Iran and Tehran Universities of Medical Sciences. Students who decided not to participate in our study were excluded. The questionnaire data for this study was gathered during the period extending from October 25, 2022, to December 11, 2022. In this study, we obtained verbal consent from all participants before they completed the questionnaire. If any participants expressed disinterest or chose not to participate, we respected their decision and did not collect any data from them.

### Tests of psychometric properties and statistical analyses

#### Face validity

The face validity of the Persian version of the EBMQ was assessed through qualitative method. To check the face validity, 5 experts, including professors, researchers in the field of evidence-based medicine, and experts in methodology and statistics, commented qualitatively about the ease of questions, grammatical errors, and lack of ambiguity, which were approved as a whole [23].

#### Content validity

To check the content validity, 15 experts and students qualified to participate in the sample commented on the importance and necessity of each questionnaire item. The calculation of content validity was done quantitatively by calculating the indexes of content validity ratio (CVR) and content validity index (CVI). Experts were asked to rate the necessity of each item using a 3-part Likert scale.

The CVR was calculated using the formula: CVR = [ne—(N/2)] / (N/2) (eq. 1), where ne represents the number of experts indicating that the item is essential and n refers to the total number of experts. Items with a CVR higher than 0.62 were retained, while those with lower scores were removed. The Content Validity Index (CVI) was also measured by asking experts about the relevance of each question using a 4-part Likert scale. The CVI was calculated using the formula: CVI = number of answers 3 and 4 / number of experts. Questions with CVI higher than 0.79 were kept, those with CVI in the range of 0.7–0.79 were revised, and questions with CVI lower than 0.7 were excluded.

To establish the structural validity of the scale, exploratory factor analysis (EFA) followed by confirmatory factor analysis (CFA) was performed. The adequacy of the EFA was determined using Bartlett’s sphericity test and the Kaiser-Mayer-Olkin (KMO) measure of sampling adequacy (good if ≥0.6). For PCA-based factor extraction, the scree plot was examined, and factors with eigenvalues >1 were extracted and Factors with factor loadings ≥0.3 were considered for interpretation. In cases where an item loaded on multiple factors, the factor with the highest loading was considered the owner of that item.

Various CFA models were compared using χ2/df, Tucker-Lewis Index (TLI), Comparative Fit Index (CFI), Normed Fit Index (NFI), and Root Mean Square Error of Approximation (RMSEA). Good model fit was indicated by values less than 4 for χ2/df, values >0.9 for CFI, NFI, and TLI, and values ≤0.06 for RMSEA [24].

The internal consistency of the Persian version of the EBMQ was assessed by calculating the pre- and post-test Cronbach’s α coefficient (acceptable if α≥0.7) [25]. Two-week test-retest reliability for each domain was evaluated using the Intra-class Correlation Coefficient (ICC). The data were analyzed using IBM SPSS v.24 and AMOS v.24. significant level was considered 5%.

### Ethics approval and consent to participate

This study was part of MSc thesis at Tehran University of Medical Sciences Ethics committee approval was received from Tehran University of Medical Sciences (IR.TUMS.SPH.REC.1400.358). Verbal informed consent was obtained from all participants.

## Results

### Translation and cross-cultural adaptation

The face validity of the Persian version of the EBMQ was confirmed by five experts. The experts agreed that the questionnaire items were appropriate. The questionnaire includes experts’ comments on replacing some words. The EBMQ consists of 42 main items in the knowledge, practice, and barrier domains. The content validity of the 42 items in the Persian version of the EBMQ was assessed based on the opinions of 15 experts. For 40 items, the CVR earned scores higher than 0.62, and the CVI for these items was calculated to be 1.0, indicating good content validity. In the knowledge domain, the CVR for two items was calculated as -0.33 and -0.6. These items were subsequently removed.

### Respondent characteristics

In this study, a total of 400 medical students engaged in various stages of their training, such as internship, residency, specialty, fellowship, and subspecialty, were included as respondents. The demographic information of the participants can be found in Table 1.

**Table 1:** Demographic characteristics of participants.

Variable	levels	N(%)
Gender	Female	211(52.8%)
	Male	189 (47.3%)
Age groups	23–27 years old	96 (24%)
	28–32 years old	174 (43.5%)
	33–37 years old	84 (21%)
	38 years old and above	46 (11.5%)
Educational level	Internship	55 (13.8%)
	Specialty	296 (74%)
	Fellowship	18 (4.5%)
	Sub-Specialty	31 (7.8%)
Study Place	Tehran University of Medical Sciences	148 (37%)
	Shahid Beheshti University of Medical Sciences	150 (37.5%)
	Iran University of Medical Sciences	102 (25.5%)

### Psychometric validation

**Fig 1:**
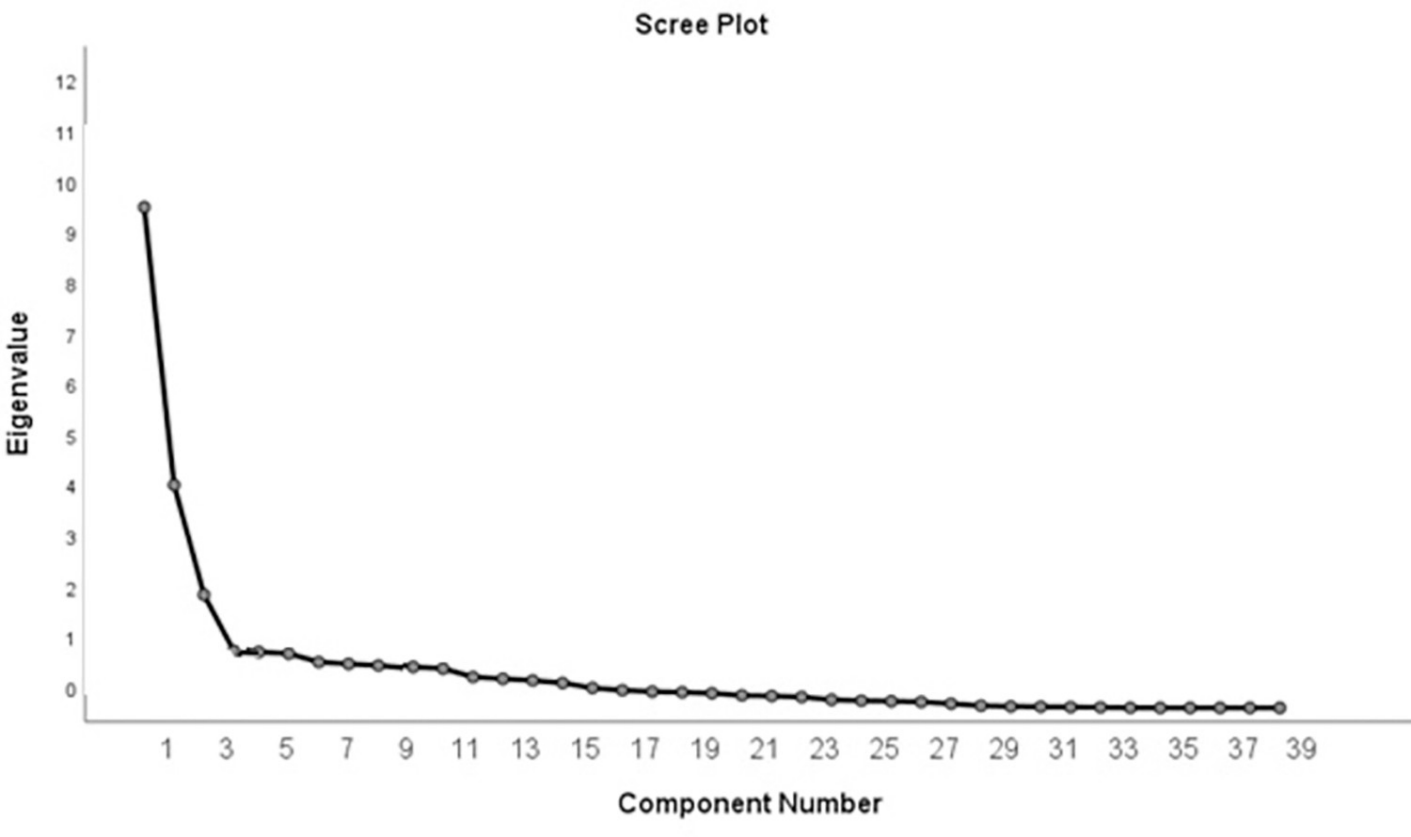
Scree plot derived from exploratory factor analysis.

The Cronbach’s α coefficients for each factor and the overall scale, as presented in Table 2, demonstrate a level of internal consistency that surpasses mere satisfaction. Additionally, the 2-week test-retest reliability of the EBMQ was deemed acceptable (ICC = 0.909, 95% CI: 0.866 to 0.941). Structural validity was assessed through exploratory factor analysis (EFA). The KMO value was 0.862, and the results of Bartlett’s test were significant (p<0.001), indicating the suitability for conducting factor analysis. Based on the scree plot (Fig 1) and the results of the EFA, three common factors with eigenvalues >1 were identified, collectively accounting for 51.61% of the total variance. Consequently, the scale items were categorized into three subscales. The EFA results and the variance explained by each factor are presented in Table 2, which shows that the three factors, namely knowledge, practice, and barriers, were consistent with the original version.

**Table 2:** The results of the exploratory factor analysis.

**Subscales EBMQ Item**	**Factors**			
	**Knowledge**	**Practice**	**Barrier**	
**C. Please indicate those which you are aware of and have used in clinical decision making?**				
C1: Aware of Evidence Based Medicine (from BMJ publishing group)	0.737		0.118	
C2: Aware of Database of abstracts of reviews of effectiveness (DARE)	0.739		0.163	
C3: Aware of Centre of Evidence-based medicine (CEBM)	0.671			
C4: Aware of ACP Journal Club	0.699	0.157		
C5: Aware of BMJ Clinical Evidence	0.672	0.124		
C6: Aware of Centre of Reviews & Dissertation	0.566			
C7: Aware of Systematic review	0.751		0.121	
C8: Aware of Meta-analysis	0.754		0.166	
C9: Aware of Case-control study	0.707			
C10: Aware of Randomized controlled trial	0.713	0.162		
C11: Aware of Relative risk	0.685	0.129		
C12: Aware of Absolute risk	0.565			
C13: Aware of Odds ratio	0.760		0.122	
C14: Aware of P-value	0.764		0.167	
C15: Aware of Level of evidence	0.738			
C16: Aware of Number needed to treat	0.733	0.142		
C17: Aware of Confidence interval	0.694	0.123		
C18: Aware of Heterogeneity	0.611			
C19: Aware of Publication bias	0.688		0.115	
C20: Aware of Test sensitivity and specificity	0.697		0.179	
C21: Aware of Positive predictive value	0.700	0.104		
C22: Aware of Clinical effectiveness	0.652	0.145		
**D. What is your opinion regarding EBM?**				
D1: I support EBM	0.102	0.811		
D2: I trust the findings from research studies		0.819		
D3: Reading research papers is important to me		0.809	0.114	
D4: EBM improves my patient care	0.101	0.815		
D5: EBM reduces my workload		0.521	0.144	
D6: I can implement EBM in my clinical practice		0.676	0.169	
D7: EBM guides my clinical decision making		0.809	0.123	
D8: I prefer to manage patients based on EBM		0.805	0.103	
**E. What difficulties do you face when practicing evidence-based medicine?**				
E1: I am able to assess the quality of research.	0.199	0.232	0.423	
E2: I have access to internet to practice EBM		0.216	0.645	
E3: I have time to read research papers.			0.714	
E4: I have time to practice EBM in my clinic			0.716	
E5: My clinic facilities are adequate to support the practice of EBM.	-0.129		0.736	
E6: Research articles are easily available to me.			0.763	
E7: My patient prefers me to practice EBM		0.210	0.614	
E8: My patient believes in information that is based on evidence	0.115	0.192	0.648	
E9: My colleagues support the practice of EBM.	0.157	0.228	0.578	
E10: My organization supports the practice of EBM.			0.703	
**Eigenvalues**	11.261	5.776	3.608	
**Variance explained (%)**	28.151	14.440	9.019	
**Cumulative Variance explained (%)**	27.178	39.911	51.61	
**Reliability: Cronbach’s α**	0.944	0.914	0.941	Overall = 0.957

A confirmatory factor analysis (CFA) was conducted to validate the structure proposed by the EFA. The results indicated that the study data best aligned with three factors consisting of 40 items. The CFA results, along with standardized parameter estimates, are presented in Table 3. In the Persian version of the EBMQ (S1 Appendix), we only assessed the validity and reliability of the 40 main items.

**Table 3:** The results of confirmatory factor analysis.

Observed variables	Latent construct	Standardized parameter estimates	p
C. Please indicate those which you are aware of and have used in clinical decision making?			
C1: Aware of Evidence Based Medicine (from BMJ publishing group)	Knowledge	0.52	0.008
C2: Aware of Database of abstracts of reviews of effectiveness (DARE)		0.61	0.012
C3: Aware of Centre of Evidence-based medicine (CEBM)		0.73	0.001
C4: Aware of ACP Journal Club		0.52	0.020
C5: Aware of BMJ Clinical Evidence		0.57	0.015
C6: Aware of Centre of Reviews & Dissertation		0.54	0.001
C7: Aware of Systematic review		0.91	0.001
C8: Aware of Meta-analysis		0.83	0.031
C9: Aware of Case-control study		0.71	0.027
C10: Aware of Randomized controlled trial		0.36	0.022
C11: Aware of Relative risk		0.42	0.013
C12: Aware of Absolute risk		0.48	0.001
C13: Aware of Odds ratio		0.53	0.005
C14: Aware of P-value		0.68	0.028
C15: Aware of Level of evidence		0.49	0.018
C16: Aware of Number needed to treat		0.38	0.001
C17: Aware of Confidence interval		0.60	0.003
C18: Aware of Heterogeneity		0.54	0.001
C19: Aware of Publication bias		0.36	0.021
C20: Aware of Test sensitivity and specificity		0.77	0.015
C21: Aware of Positive predictive value		0.76	0.001
C22: Aware of Clinical effectiveness		0.67	0.035
**D. What is your opinion regarding EBM?**			
D1: I support EBM	**Practice**	0.59	0.001
D2: I trust the findings from research studies		0.49	0.027
D3: Reading research papers is important to me		0.88	0.018
D4: EBM improves my patient care		0.64	0.018
D5: EBM reduces my workload		0.45	0.016
D6: I can implement EBM in my clinical practice		0.58	0.022
D7: EBM guides my clinical decision making		0.52	0.008
D8: I prefer to manage patients based on EBM		0.47	0.001
**E. What difficulties do you face when practicing evidence-based medicine?**			
E1: I am able to assess the quality of research.	Barrier	0.87	0.001
E2: I have access to internet to practice EBM		0.87	0.015
E3: I have time to read research papers.		0.65	0.013
E4: I have time to practice EBM in my clinic		0.70	0.018
E5: My clinic facilities are adequate to support the practice of EBM.		0.91	0.015
E6: Research articles are easily available to me.		0.58	0.022
E7: My patient prefers me to practice EBM		0.57	0.008
E8: My patient believes in information that is based on evidence		0.86	0.001
E9: My colleagues support the practice of EBM.		0.60	0.001
E10: My organization supports the practice of EBM.		0.83	0.018

Fit indices: Chi-square statistics/df = 4.23; RMSEA = 0.08; CFI = 0.95; NFI = 0.93; TLI = 0.92.

## Discussion

The EBMQ was originally developed to measure knowledge, practice, and barriers to EBM in medical care in Malaysia and proved valid and reliable [17]. We translated and cross‐culturally adapted the EBMQ into a Persian version following international guidelines [18,20,21] and assessed its validity and reliability with an adequate sample size. Our findings support the Persian version of the EBMQ as a valid and reliable tool.

A phase of this research involved translating an EBMQ into Persian. This task was undertaken by individuals proficient in English and experienced in EBM concepts, who translated the questionnaire in both directions. The final version underwent a review by a committee of experts, and their feedback was incorporated. This process resulted in the preparation of the final questionnaire. Habibi et al. (2022) conducted a study titled “Reliability and Validity of the Persian Version of the ACE Tool: Assessing Medical Trainees’ Competency in Evidence-Based Medicine”. In this study, the process used for translating the ACE tool was similar to the one employed in the current study[26]. We discovered that another group was simultaneously translating and validating EBMQ into Persian and their study population was Persian Medicine (PM) specialists [27]. However, Hashem Dabaghian et al. (2022) initially, the original questionnaire translated into Persian. Subsequently, an English-fluent individual re-translated this Persian version back into English. The final step involved comparing this re-translated English version with the original questionnaire to ensure accuracy [27]. It’s worth noting that the translation methodology employed in this research differed from that used in the current study.

The content validity of the Persian version of the EBMQ was calculated using CVR and CVI. The original EBMQ consists of 42 main items. The scores were desirable for 40 items [28], and two items were removed from the knowledge domain. In the current study to determine the construct validity of the translated EBMQ, both exploratory factor analysis and confirmatory factor analysis were used. The results of EFA showed that the translated EBMQ had a three‐factor structure that accounted for 51.61% of the total variance. Based on the results, the Persian version of the EBMQ matched the original instrument [17]. The results of CFA indicated, that all variables had a good fit to the respective construct. Exploratory factor analysis and confirmatory factor analysis demonstrated the construct validity of the translated EBMQ.

Habibi et al. (2022) in their study performed a reliability and validity evaluation of the Persian version of the ACE tool. They assessed face validity and content validity, utilizing the CVR and CVI indices, with the assistance of 15 faculty members and the Lawshe method. Additionally, they employed confirmatory factor analysis to investigate the validity of the factor structure [26]. In 2021, Cakmakkaya et al. conducted a study titled “Cross-Cultural Adaptation of Fresno Test for Turkish Language”. In this study, a group of five physicians, all experts in EBM, examined the content validity of the questionnaire. They employed the Lawshe technique for this evaluation, and calculated the Content Validity Ratio (CVR) and Content Validity Index (CVI) to quantify the content validity [29]. The approach they used to assess content validity shares resemblance to the method used in our current study. In the study conducted by Hashem-Dabaghian et al. [27] the same methods were implied for evaluating CVI and CVR, but none of the items in their study were removed. They used best practices in calculating face and content validity. Construct validity have not been addressed in their research because of inadequate sample size.

The internal consistency reliability test determines how all factors on the test relate to all other factors. Cronbach’s α is the most commonly used internal consistency measure [30]. In this study, we calculated Cronbach’s α to determine internal consistency. Overall Cronbach’s α (0.957) and for each domain (0.914 to 0.944) were satisfactory. These values indicated the acceptable reliability of the Persian version of the EBMQ. The results of internal consistency and two-week test‐retest reliability in this study were close to the results of the original EBMQ [17]. Zhou et al. [5] in the study entitled “Translation, cultural adaptation, validation, and reliability study of the Quick‐EBP‐VIK instrument: Chinese version” calculated Cronbach’s α to determine internal consistency. The calculated Cronbach’s α in this study was reported to be higher than 0.7. The method of determining internal consistency in their research was similar to that in the present study. Hashem Dabaghian et al. (2022), Habibi et.al (2022) and Cakmakkaya et al. (2021) for internal consistency were also used the Cronbach’s alpha.

This study was conducted among postgraduate medical students in Iran. Our findings showed that, although all participants were familiar with EBM and the majority of them had completed training courses, most of them were not familiar with EBM databases. As the findings of a number of previous studies have revealed [10,27], the findings of this study also showed, that workload and lack of time, limited access to the Internet and lack of access to suitable facilities for applying EBM at work were some of the main barriers to EBM implementation. This means that more efforts should be made to facilitate the implementation of EBM among the medical community. Tohidast et al. (2021) pointed out these barriers in their qualitative study about the challenges of implementing evidence-based practice among Iranian speech and language pathologists [31]. Ghojazadeh et al. (2015) in their study entitled “A systematic review on barriers, facilities, knowledge and attitude toward evidence-based medicine in Iran”, demonstrated that the most significant barriers to EBM were a lack of facilities, time, and skills in research methodology [10].

Directions for future research include applying the Persian version of EBMQ among different populations, conducting studies that assess the effect of EBM training on the knowledge and practice of health-care providers, and conducting research that evaluates the main barriers in EBM in Iran.

The findings of this study hold significant implications for the implementation of EBM in Iran. EBMQ serves as a tool to pinpoint areas of deficiency in knowledge and practice among Persian physicians, thereby facilitating the creation of specific interventions to enhance care quality. Furthermore, employing the EBMQ can foster an environment of evidence-based practice within the Iranian healthcare professional community.

This study has several strengths. First, we used a rigorous translation and cross-cultural adaptation process to ensure the validity and reliability of the Persian version of the EBMQ. Additionally, we evaluated the psychometric properties of the EBMQ among a large sample of postgraduate medical students in Iran. Furthermore, we applied a variety of statistical methods to evaluate the reliability and validity of the EBMQ.

### Limitations

One of the limitations of this study was that this research was conducted among medical students studying in internship, residency, specialty, fellowship and subspecialty at the three most prestigious universities in Iran; therefore, the generalizability of this study is limited.

## Conclusions

The psychometric analysis of the Persian version of the EBMQ indicates that this tool demonstrates acceptable reliability and validity. It can serve as a valuable instrument for measuring knowledge, practice, and barriers related to EBM in Iranian healthcare settings. Those responsible for medical education in medical sciences universities can utilize the items encompassing all three dimensions of this questionnaire to enhance EBM courses.

## Supporting information

S1 Appendix Persian version of Evidence Based Medicine Questionnaire (EBMQ). (DOCX)
