# Supplementary material for: Translation and cross-cultural adaptation of Persian version of Evidence Based Medicine Questionnaire (EBMQ) in postgraduate medical students in Iran
Source: PLoS One. 2024 Apr 16;19(4):e0301831. doi: 10.1371/journal.pone.0301831 (PMC11020528; doi:10.1371/journal.pone.0301831)
Supplement: S1 Appendix — (DOCX) [file pone.0301831.s001.docx]

**Supplementary appendix 1**: Persian Version of Evidence Based Medicine Questionnaire (EBMQ).

پرسشنامه پزشکی مبتنی بر شواهد(EBMQ)

**بخش اول: مشخصات دموگرافیک**

1- جنسیت: مرد  زن

2- سن(سال):

3- آخرین مدرک تحصیلی:

کارورز (اینترن)  تخصص بالینی  فلوشیپ  فوق تخصص

4- محل اشتغال به کار یا تحصیل:

دانشگاه علوم پزشکی تهران و مراکز وابسته به آن

دانشگاه علوم پزشکی شهید بهشتی و مراکز وابسته به آن

دانشگاه علوم پزشکی ایران و مراکز وابسته به آن

**بخش دوم: منابع اطلاعاتی**

5- بطور میانگین هر چند وقت یکبار اطلاعات بالینی را از متون پزشکی جستجو می کنید؟

(جستجو می تواند از کتابهای درسی، نشریات علمی یا پایگاههای اطلاعاتی آنلاین باشد)

همیشه (چندین بار در هفته)  اغلب اوقات (یکبار در هفته)  گاهی اوقات (حداقل یکبار در ماه)

به ندرت (چندماه یکبار)  در یکسال گذشته هرگز

6- در یک سال گذشته، چند مرتبه اطلاعات پزشکی را از منابع زیر جستجو کرده‌اید؟

(برای هر آیتم، لطفا یک پاسخ را که به بهترین نحو وضعیت شما را نشان می دهد علامت بزنید.)

| **منابع اطلاعاتی** | **همیشه**  **(چندین بار در هفته)** | **اغلب**  **(یکبار در هفته)** | **گاهی اوقات**  **(حداقل یکبار در ماه)** | **به ندرت**  **(چند ماه یکبار)** | **در یک سال گذشته هرگز** | **عدم دسترسی** |
| --- | --- | --- | --- | --- | --- | --- |
| کتب درسی |  |  |  |  |  |  |
| مقالات مجلات |  |  |  |  |  |  |
| گایدلاین‌های بالینی |  |  |  |  |  |  |
| پایگاه‌های اطلاعاتی آنلاین(مانند: PubMed، کاکرین و پایگاه TRIP) |  |  |  |  |  |  |
| وب سایت‌های پزشکی (مانند:  (Medscape, MedlinePlus, Web Med) |  |  |  |  |  |  |
| موتورجستجو و منابع اطلاعاتی عمومی (مانند: Google, Wikipedia ) |  |  |  |  |  |  |
| شبکه های اجتماعی (مانند: WhatsApp,  Telegram, Instagram, Facebook) |  |  |  |  |  |  |
| اپلیکیشن‌های پزشکی (مانند: ePocrates, Medical Calculator) |  |  |  |  |  |  |
| همتایان/ همکاران |  |  |  |  |  |  |
| کنفرانس‌ها/  سخنرانی‌ها/ سمینارها/ ژورنال کلاب |  |  |  |  |  |  |

7- آیا اصطلاح "پزشکی مبتنی بر شواهد" را شنیده اید؟

بله  خیر

8- آیا تا به حال در یک دوره آموزشی یا کارگاه پزشکی مبتنی بر شواهد شرکت داشته اید؟

بله  خیر

9- آیا تا به حال آموزش رسمی در حوزه های زیر دریافت کرده‌اید؟

* فرموله‌بندی و تدوین سؤال پژوهش بله  خیر

* جستجوی متون بله  خیر

* ارزیابی نقادانه بله  خیر

**بخش سوم: دانش و عملکرد پزشکی مبتنی بر شواهد**

10- در این بخش برخی از منابع موجود و قابل استفاده در بکارگیری پزشکی مبتنی بر شواهد آمده است. لطفاً مشخص نمایید با کدامیک از این منابع آشنا هستید و در تصمیم‌گیری‌های بالینی بکار گرفته‌اید.

(برای هر آیتم، لطفا یک پاسخ را که به بهترین نحو وضعیت شما را نشان می دهد علامت بزنید.)

| **منابع اطلاعاتی** | **بی اطلاع** | **اطلاع دارم اما در تصمیم‌گیری بالینی استفاده نکرده‌ام** | **در مورد آن خوانده ام اما در تصمیم گیری‌ بالینی استفاده نکرده‌ام** | **خوانده‌ام و در تصمیم‌گیری بالینی استفاده کرده‌ام** |
| --- | --- | --- | --- | --- |
| Evidence Based Medicine (از گروه انتشاراتی BMJ) |  |  |  |  |
| Database of abstracts of reviews of effectiveness (DARE) |  |  |  |  |
| Centre of Evidence-Based Medicine (CEBM) |  |  |  |  |
| ACP Journal Club |  |  |  |  |
| BMJ Clinical Evidence |  |  |  |  |
| Centre of Reviews & Dissertation |  |  |  |  |

11- در زیر اصطلاحاتی که معمولاً در پزشکی مبتنی بر شواهد استفاده می‌شود آورده شده است.

(برای هر آیتم، لطفاً یک پاسخ را که به بهترین نحو وضعیت شما را نشان می‌دهد علامت بزنید.)

| **اصطلاحات** | **این اصطلاح را اصلا نشنیده‌ام** | **این اصطلاح را شنیده‌ام اما معنی آن را نمی‌دانم** | **این اصطلاح را نمی‌دانم اما علاقه‌مندم درباره آن بدانم** | **تا حدودی با مفهوم این اصطلاح آشنا هستم** | **به خوبی این اصطلاح را می‌دانم و می‌توانم مفهوم آن را برای دیگران شرح دهم** |
| --- | --- | --- | --- | --- | --- |
| Systematic review (مرور نظام‌مند) |  |  |  |  |  |
| Meta-analysis (فراتحلیل، متاآنالیز) |  |  |  |  |  |
| Case-control study (مطالعه مورد-شاهدی) |  |  |  |  |  |
| Randomized controlled trial (کارآزمایی بالینی تصادفی شده) |  |  |  |  |  |
| Relative risk (خطر نسبی) |  |  |  |  |  |
| Absolute risk (خطر مطلق) |  |  |  |  |  |
| Odds ratio (نسبت شانس) |  |  |  |  |  |
| P-value (پی ولیو) |  |  |  |  |  |
| Level of evidence (سطح شواهد) |  |  |  |  |  |
| Number needed to treat (تعداد مورد نیاز برای درمان) |  |  |  |  |  |
| Confidence interval  (فاصله اطمینان) |  |  |  |  |  |
| Heterogeneity  (ناهمگنی) |  |  |  |  |  |
| Publication bias  (سوء گیری انتشار) |  |  |  |  |  |
| Test sensitivity and specificity  (آزمون حساسیت و ویژگی) |  |  |  |  |  |
| Positive predictive value  (ارزش اخباری مثبت) |  |  |  |  |  |
| Clinical effectiveness  (اثربخشی بالینی) |  |  |  |  |  |

12- نظر شما در مورد پزشکی مبتنی بر شواهد چیست؟ (برای هر آیتم، لطفاً یک پاسخ را که به بهترین نحو وضعیت شما را نشان می‌دهد علامت بزنید.)

| **گویه‌ها** | **کاملاً مخالف** | **مخالف** | **نه موافق نه مخالف** | **موافق** | **کاملاً موافق** |
| --- | --- | --- | --- | --- | --- |
| از پزشکی مبتنی بر شواهد حمایت می کنم. |  |  |  |  |  |
| به یافته‌های مطالعات پژوهشی اعتماد دارم |  |  |  |  |  |
| مطالعه مقالات پژوهشی برای من اهمیت دارد |  |  |  |  |  |
| پزشکی مبتنی بر شواهد باعث بهبود مراقبت از بیمارم شده است |  |  |  |  |  |
| پزشکی مبتنی بر شواهد حجم کاری مرا کم کرده است |  |  |  |  |  |
| من می‌توانم پزشکی مبتنی بر شواهد را در عملکرد بالینی خود بکار گیرم |  |  |  |  |  |
| پزشکی مبتنی بر شواهد راهنمای تصمیم‌گیری بالینی من است |  |  |  |  |  |
| من ترجیح می دهم مدیریت بیماران را بر اساس پزشکی مبتنی بر شواهد پیش ببرم |  |  |  |  |  |

**بخش چهار: موانع و تسهیلات عملکرد مبتنی بر شواهد**

13- در اینجا برخی از مشکلاتی که ممکن است هنگام بکارگیری پزشکی مبتنی بر شواهد با آن روبرو شوید آورده شده است. (برای هر آیتم، لطفاً یک پاسخ را که به بهترین نحو وضعیت شما را نشان می‌دهد علامت بزنید.)

| **گویه‌ها** | **کاملاً مخالف** | **مخالف** | **نه موافق نه مخالف** | **موافق** | **کاملاً موافق** |
| --- | --- | --- | --- | --- | --- |
| من قادرم کیفیت پژوهش را ارزیابی کنم |  |  |  |  |  |
| برای بکارگیری پزشکی مبتنی بر شواهد به اینترنت دسترسی دارم |  |  |  |  |  |
| برای مطالعه مقالات پژوهشی زمان دارم |  |  |  |  |  |
| در محل کارم زمان کافی برای بکارگیری پزشکی مبتنی بر شواهد دارم |  |  |  |  |  |
| امکانات محل کار من برای بکارگیری پزشکی مبتنی بر شواهد کافی است |  |  |  |  |  |
| مقالات پژوهشی به آسانی برای من قابل دسترس هستند |  |  |  |  |  |
| بیمارم ترجیح می‌دهد پزشکی مبتنی بر شواهد را بکار ببرم |  |  |  |  |  |
| بیمارم به اطلاعاتی که مبتنی بر شواهد است اعتماد دارد |  |  |  |  |  |
| همکاران من از به کارگیری پزشکی مبتنی بر شواهد حمایت می کنند. |  |  |  |  |  |
| سازمان من از به کارگیری پزشکی مبتنی بر شواهد حمایت می‌کند |  |  |  |  |  |

* چنانچه در زمینه پزشکی مبتنی بر شواهد پیشنهادی دارید لطفاً ذکر نمایید.
